# Supplementary material for: Trait correlates of distribution trends in the Odonata of Britain and Ireland
Source: PeerJ. 2015 Nov 19;3:e1410. doi: 10.7717/peerj.1410 (PMC4655099; doi:10.7717/peerj.1410)
Supplement: Supplemental Information 1 — Appendix 1. Detailed description of the Bayesian occupancy model used to estimate trends in species’ occurrence. Appendix 2. The posterior distribution of the trend estimates for each species are summarised here as the mean, standard deviation and the lower 2.5 and upper 97.5 percentile. All values were rounded to 4 decimal places. These data alongside the trait data (freely available here: doi: 10.3897/BDJ.2.e1041) form the raw data for comparative trait analysis that this study is based upon. [file peerj-03-1410-s001.docx]

**Supplementary information**

**Appendix 1**. Detailed description of the Bayesian occupancy model used to estimate trends in species’ occurrence

The Bayesian occupancy model approach was used to estimates true occupancy per site per year given imperfect detection. Occupancy models require detection/non-detection data during replicated visits within a closure period. In our model, the closure period spans a single year. Non-detection was inferred for each species not recorded on a visit where at least one other species was recorded. The model is run for each species separately but uses data from other species to infer non-detection and inform detection probability (described below).

The occupancy model consists of two hierarchically coupled sub-models, one, the state model, governs the true presence/absence of a species at a site in a given year, the second, the observation model, governs the probably of detecting that species given its presence or absence, and is therefore conditional on the state model (Appendix 1.1). For each site year combination the model estimates presence or absence for the species in question (*z_it_*), which is linked to the observed data (y*_itv_*), given variation in detection probability (p*_itv_*). These *z_it_* values were then combined to create an annual estimate of the proportion of occupied sites, which was in turn regressed against year to estimate a temporal trend in species’ occurrence.

The detection probability is estimated following capture-recapture theory (MacKenzie, 2006; van Strien *et al.*, 2013) and is informed by the number of species recorded on a given visit (*L_itv_*), a proxy for sampling effort. The full model equation can be seen in Appendix 1.1 and the model structure is illustrated in Appendix 1.2. Non-informative priors are used throughout these models

MacKenzie, D.I. (2006) *Occupancy Estimation and Modeling: Inferring Patterns and Dynamics of Species Occurrence*, Academic Press, Burlington, Massachusetts, USA.

Van Strien, A.J., van Swaay, C.A.M. & Termaat, T. (2013) Opportunistic citizen science data of animal species produce reliable estimates of distribution trends if analysed with occupancy models. *Journal of Applied Ecology*, **50**, 1450–1458.

**Appendix 1.1:** The Bayesian occupancy model used to estimate annual site occupancy.

*State model* - z*_it_* ~ Bernoulli(*ψ_it_*); logit(*ψ_it_*) = *b_t_* + *u_i_*

*Observation model* - y*_itv_*|z*_it_* ~ Bernoulli(z*_it_* * p*_itv_*); logit (p*_itv_*) = *a_t_* + *c*.log(L*_itv_*)

*z_it_* = True occupancy of site (i) in year (t). Can be a 1 or 0, present or absent.

*ψ_it_* = The probability that site (i) is occupied in year (t)

*b_t_* = Year effect (categorical)

*u_i_* = Site effect (categorical)

y*_itv_* = Observed presence/absence at site (i) at year (t) on visit (v)

p*_itv_* = The probability of detection at site (i) at year (t) on visit (v), conditional on *Z_it_* that is the species true presence or absence.

*a_t_* = Year level random effect (categorical)

*L_itv_* = List length at site (i) in year (t) on visit (v)

*c* = Change in the log-odds of detectability associated with an increasing list length by a factor of *e.*

**Appendix 1.2** Directed acyclic graph illustrating the occupancy model structure. Orange shading represents the state model, blue shading represents the observation model, and the green box represents the observed data.


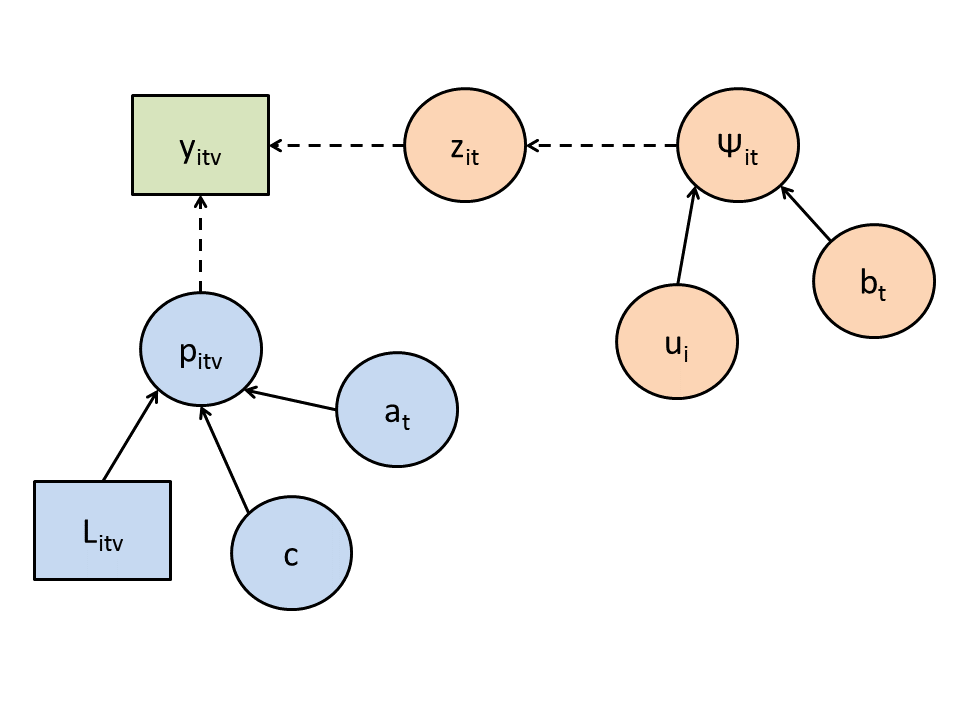


**Appendix 2** The posterior distribution of the trend estimates for each species are summarised here as the mean, standard deviation and the lower 2.5 and upper 97.5 percentile. All values were rounded to 4 decimal places.

| **Species** | **Trend** | **Trend sd** | **Trend 2.5** | **Trend 97.5** |
| --- | --- | --- | --- | --- |
| Aeshna caerulea | -0.0008 | 0.0015 | -0.0060 | 0.0000 |
| Aeshna cyanea | 0.0021 | 0.0007 | 0.0007 | 0.0035 |
| Aeshna grandis | 0.0049 | 0.0005 | 0.0038 | 0.0060 |
| Aeshna juncea | -0.0033 | 0.0005 | -0.0044 | -0.0023 |
| Aeshna mixta | 0.0112 | 0.0007 | 0.0097 | 0.0126 |
| Anax imperator | 0.0147 | 0.0008 | 0.0131 | 0.0163 |
| Brachytron pratense | 0.0047 | 0.0004 | 0.0039 | 0.0054 |
| Calopteryx splendens | 0.0058 | 0.0004 | 0.0051 | 0.0066 |
| Calopteryx virgo | 0.0011 | 0.0003 | 0.0004 | 0.0018 |
| Ceriagrion tenellum | -0.0003 | 0.0002 | -0.0007 | 0.0001 |
| Coenagrion hastulatum | -0.0001 | 0.0000 | -0.0002 | 0.0000 |
| Coenagrion mercuriale | 0.0001 | 0.0000 | 0.0000 | 0.0001 |
| Coenagrion puella | 0.0002 | 0.0006 | -0.0010 | 0.0014 |
| Coenagrion pulchellum | -0.0001 | 0.0003 | -0.0007 | 0.0004 |
| Cordulegaster boltonii | -0.0009 | 0.0004 | -0.0017 | -0.0001 |
| Cordulia aenea | 0.0008 | 0.0002 | 0.0004 | 0.0012 |
| Enallagma cyathigerum | 0.0013 | 0.0004 | 0.0004 | 0.0021 |
| Erythromma najas | 0.0082 | 0.0004 | 0.0074 | 0.0090 |
| Gomphus vulgatissimus | -0.0004 | 0.0001 | -0.0007 | -0.0002 |
| Ischnura elegans | 0.0017 | 0.0003 | 0.0011 | 0.0023 |
| Ischnura pumilio | -0.0020 | 0.0003 | -0.0025 | -0.0015 |
| Lestes dryas | -0.0002 | 0.0009 | -0.0041 | 0.0002 |
| Lestes sponsa | -0.0028 | 0.0007 | -0.0041 | -0.0014 |
| Leucorrhinia dubia | -0.0002 | 0.0001 | -0.0003 | -0.0001 |
| Libellula depressa | 0.0056 | 0.0008 | 0.0041 | 0.0072 |
| Libellula fulva | 0.0014 | 0.0001 | 0.0011 | 0.0016 |
| Libellula quadrimaculata | 0.0048 | 0.0007 | 0.0035 | 0.0062 |
| Orthetrum cancellatum | 0.0099 | 0.0007 | 0.0084 | 0.0112 |
| Orthetrum coerulescens | 0.0004 | 0.0002 | -0.0001 | 0.0008 |
| Platycnemis pennipes | 0.0026 | 0.0002 | 0.0021 | 0.0030 |
| Pyrrhosoma nymphula | 0.0057 | 0.0005 | 0.0046 | 0.0067 |
| Somatochlora arctica | 0.0004 | 0.0007 | -0.0002 | 0.0016 |
| Somatochlora metallica | -0.0002 | 0.0002 | -0.0007 | 0.0001 |
| Sympetrum danae | -0.0029 | 0.0004 | -0.0036 | -0.0021 |
| Sympetrum sanguineum | 0.0105 | 0.0008 | 0.0089 | 0.0121 |
| Sympetrum striolatum | 0.0000 | 0.0008 | -0.0017 | 0.0014 |
